# Dietary Isoleucine and Valine: Effects on Lipid Metabolism and Ureagenesis in Pigs Fed with Protein Restricted Diets

**DOI:** 10.3390/metabo13010089

**Published:** 2023-01-05

**Authors:** Parniyan Goodarzi, Mohammad Habibi, Matthew William Gorton, Katherine Walsh, Firoozeh Tarkesh, Mallory Fuhrig, Adel Pezeshki

**Affiliations:** Department of Animal and Food Sciences, Oklahoma State University, Stillwater, OK 74078, USA

**Keywords:** very low protein diet, valine, isoleucine, ureagenesis, lipid metabolism

## Abstract

A mixture of valine (Val) and isoleucine (Ile) not only decreases the negative impact of very low protein (VLP) diets on the growth of pigs, but also influences the nitrogen (N) balance and lipid metabolism; however, the underlying pathways are not well understood. This study aimed to investigate the effect of dietary Val and Ile on lipogenesis, lipolysis, and ureagenesis under protein restriction. After one week of acclimation, forty three-week-old pigs were randomly assigned to following dietary treatments (*n* = 8/group) for 5 weeks: positive control (PC): normal protein diet; negative control (NC): VLP diet; HV: NC supplemented with Val; HI: NC supplemented with Ile; and HVI: NC supplemented with both Val and Ile. HVI partially improved the body weight and completely recovered the feed intake (FI) of pigs fed with NC. HVI increased thermal radiation and improved the glucose clearance. HVI had a lower blood triglyceride than PC and blood urea N than NC. NC and HV promoted lipogenesis by increasing the transcript of *fatty acid synthase* (*FAS*) in the liver and *lipoprotein lipase* (*LPL*) in adipose tissue but reducing *hormone-sensitive lipase* (*HSL*) in the liver. HVI reduced the increased rate of lipogenesis induced by the NC group through normalizing the mRNA abundance of hepatic *FAS*, *sterol regulatory element binding transcription factor 1*, and *HSL* and *LPL* in adipose tissue. NC, HV, HI, and HVI reduced the ureagenesis by decreasing the protein abundance of *carbamoyl phosphate synthetase I, ornithine transcarboxylase*, and *arginosuccinate lyase* in the liver. Overall, HVI improved the growth, FI, and glucose clearance, and decreased the rate of lipogenesis induced by VLP diets.

## 1. Introduction

Excess nitrogen (N) excreted from modern swine production has a negative impact on the environment through its contribution to acidification and eutrophication of sensitive ecosystems and odor emissions [1]. Reducing dietary crude protein (CP) by more than 4%-unit increases the N utilization [2,3,4], but growth performance is depressed even when the first four limiting amino acids (AAs), lysine (Lys), methionine (Met), threonine (Thr), and tryptophan (Trp), are supplemented in the diet of early weaned, growing, and finishing pigs [5,6,7,8].

We have previously demonstrated that supplementing very low protein (VLP) diets with both limiting AAs and branched-chain AAs (BCAAs), leucine (Leu), isoleucine (Ile), and valine (Val), or a mixture of Ile and Val not only decreases the negative impact of these diets on growth but also reduces the blood urea N (BUN) [9,10,11]. Others have shown that dietary supplementation of Val [12,13,14] and Ile [15] reduce BUN in pigs. Given a positive correlation between lower BUN and decreased N excretion in pigs [16,17], it appears that BCAAs promote N retention. There is evidence that dietary BCAAs may potentially increase the efficiency of AA and N utilization in pigs [18,19], and humans [20,21,22]. Little is understood on the mechanisms by which BCAAs influence the N balance. Nitrogen balance is the result of dynamic protein digestion, absorption, and metabolism. Dietary BCAAs improve the N utilization possibly by increasing the activity and/or secretion rate of proteolytic enzymes [23,24,25,26], upregulation of intestinal AA transporters [27,28,29] and providing N for de novo synthesis of AAs [30,31,32]. The effect of Val and Ile alone, or in combination, on expression of urea cycle enzymes is yet to be studied.

Our previous data provide evidence on the role of dietary BCAAs on lipid metabolism in pigs fed with VLP diets [9,33]. The literature on the regulatory role of BCAAs on lipid metabolism is equivocal. Evidence shows that dietary supplementation of BCAAs stimulate lipolysis and reduces fat deposition, white adipose tissue mass, and triglyceride (TG) concentration in the muscle and liver of mice [34,35,36,37]. Despite this, others have reported a lipogenic role for BCAAs [38,39,40,41,42,43,44]. Supplementation of BCAAs has been reported to increase the serum TG and fat accumulation in white adipose tissue in mice [38,39] while their restriction or deprivation promotes fat loss and reduces the organs TG content in rats, mice, and broilers [40,41,42,43,44]. While pigs have many similarities to humans in terms of metabolism, dietary habits, nutritional requirements, and nutrients interactions [45,46] and have been previously used as a model for studying AAs metabolism [47,48] and metabolic complications [49,50], little is understood on the role of BCAAs on lipid metabolism in pigs. To our knowledge no study has examined the effect of a combination of Val and Ile on lipid metabolism in pigs offered with VLP diets.

Since a high level of dietary Leu is associated with reduced growth rate and feed intake (FI) in weaned pigs [51,52], here we only focused on Ile and Val. Given the beneficial effects of combining Ile and Val on the growth of pigs fed with protein restricted diets [11,53], we hypothesized that over supplementation of both Val and Ile in VLP diets will have additive positive effects on the growth performance of weaned pigs and will alter lipid metabolism and decrease BUN by reducing urea cycle rate limiting enzymes. Therefore, the objective of this study was to investigate the effect of dietary Val and Ile on the growth performance, and gene and protein expression of key rate limiting enzymes of the urea cycle and lipid metabolism in pigs fed with VLP diets.

## 2. Materials and Methods

### 2.1. Animals and Housing

All the experimental procedures used in this study were reviewed and approved by Oklahoma State University’s Institutional Animal Care and Use Committee (IACUC-20-54). A total of forty, three-week-old, weaned barrows (Duroc sire line and Large White × Landrace dam) with an average body weight (BW) of 6.10 ± 0.62 kg were used (Seaboard, Hennessey, OK, USA). Upon arrival, animals were group housed in an environmentally controlled animal room as we previously described [11]. Feed was provided *ad libitum* and all pigs had free access to water during the study.

### 2.2. Diets and Experimental Design

After one week of adaption, pigs were weight-matched (average BW of 6.98 ± 0.80 kg) and randomly assigned to 5 dietary treatments (*n* = 8/group) for 5 weeks including: (1) positive control (PC): normal protein diet; (2) negative control (NC): VLP diet containing the first four limiting AAs, Lys, Met, Thr, and Trp, at National-Research-Council (NRC) [54] levels; (3) HV: NC containing standard ileal digestibility (SID) Val: Lys ratio of 0.75; (4) HI: NC containing SID Ile: Lys ratio of 0.60; and (5) HVI: NC containing SID Val: Lys ratio of 0.75 and SID Ile: Lys ratio of 0.60. The ratios for SID Val:Lys and Ile:Lys were based on previous literature where an improved performance for pigs fed with low protein diets was reported when the above values were used [16,55]. Using National Swine Nutrition Guide (NSNG; Version 2.1 Metric, ^©^2012 U.S. Pork Center of Excellence) and NRC recommendations [54] for animal nutritional requirements at different ranges of BW, three nursery phase diets were formulated. The nursery phase 1 (N1), phase 2 (N2), and phase 3 (N3) diets were fed on days 1–7, 8–21, and 22–42, respectively. All diets were formulated to be isocaloric by using variable amounts of corn and soybean meal. Further, L-Alanine was used to keep the NC, HV, HI, and HVI diets isonitrogenous. The amounts of other ingredients used were kept as consistent as possible. The ingredients and chemical composition of all diets are given in Table 1.

### 2.3. Growth Performance Traits

Individual FI and water intake (WI) were monitored daily. Further, FI was measured at 3, 6, 9, 12, and 24 h after feeding at 8 a.m., biweekly. Body weight and growth parameters including body length, wither height, and heart girth of all pigs were recorded weekly. Average daily feed intake (ADFI), average daily water intake (ADWI), average daily gain (ADG), average daily protein intake (ADPI), body weight gain (BWG), mean feed intake (MFI), cumulative feed intake (CFI), cumulative protein intake (CPI), water-to-feed ratio (W:F), gain-to-feed ratio (G:F), and gain-to-protein ratio (G:P) were calculated using BW, FI, and WI data.

### 2.4. Thermal Imaging

Weekly thermal images were acquired about 1 m above each pig (emissivity coefficient of 0.95) using a FLIR C2 compact thermal camera with a focal length of 1.54 mm and a thermal accuracy of ±2 °C (FLIR Systems, Boston, MA, USA). Representative thermal images for each dietary group are shown in Figure S1.

### 2.5. Feed, Blood and Tissue Samples Collection

The feed samples (about 50 g) were collected from each feed bag and pooled for each diet, during diet preparation. The samples were then stored at −20 °C until feed composition analysis. At week 6, pigs were allowed to consume their respective diets for one hour following an overnight fast (~8 h), and then blood samples were drawn from the jugular vein at baseline and then at 60 and 120 min after the meal test. The blood samples were collected in 10.0 mL serum tubes and 3.0 mL plasma tubes containing lithium heparin (BD Vacutainer^®^, Franklin Lakes, NJ, USA). Blood samples were centrifuged at 3000× *g* for 15 min at 4 °C, and serum or plasma was separated and stored at −80 °C. All pigs were euthanized using the CO_2_ asphyxiation method 120 min after the meal test, the liver, subcutaneous adipose tissue, and kidney samples were immediately extracted, snap-frozen in liquid nitrogen, and stored at −80 °C.

### 2.6. Thermal Radiation Analysis

The mean dorsal body surface temperature was obtained by drawing a rectangle on the entire back of pigs using the FLIR camera software (FLIR Research Studio software, FLIR Systems, Boston, MA, USA), as we previously described [56]. The following equation was then used to calculate the thermal radiation (W/m^2^): σε 〖Ts〗^4 − 〖Tα〗^4  where σ is the Stefan Boltzmann constant (5.67 × 10^−8^ W/m^2^K^4^), ε is the thermodynamic emissivity (0.95), T_s_ is the mean body surface temperature (kelvin), and T_α_ is the ambient temperature (kelvin).

### 2.7. Diets Composition Analysis

The chemical composition (i.e., dry matter, CP, crude fiber, calcium, and phosphorus) and AAs concentrations of the experimental diets were analyzed by ServiTech laboratories (Dodge City, KS, USA) [7,33,57] and Agricultural Experiment Station Chemical Laboratories (University of Missouri-Columbia, MO, USA) [56], respectively. The results of diet composition and AAs analysis are given in Table 2.

### 2.8. Plasma Metabolites and Urea Analysis

The concentrations of plasma glucose, TG, and cholesterol were determined by a chemistry analyzer (R404200-3, Alfa Wassermann’s Vet Axcel, West Caldwell, NJ, USA) using a calibrator (BL-442600, Multi-Analyte calibrator for Synchron CX/LX) and reagents (Carolina Liquid Chemistries Crop, Brea, CA, USA) for glucose (BL-208), TG (BL-213) and cholesterol (BL-211). Absorbance was recorded at 340 nm for glucose and at 505 nm for TG and cholesterol. QuantiChrom^TM^ Urea Assay Kit (DIUR-100, BioAssay Sytems, Hayward, CA, USA) was used to detect plasma urea concentration, according to the manufacturer’s instructions. The optical density was measured with an Epoch microplate spectrophotometer (BioTek^®^ Instruments, Inc. Highland Park, VT, USA) at a wavelength of 520 nm. The intra-assay coefficient of variation was 9.73%.

### 2.9. Reverse Transcription, and Quantitative PCR (RT-qPCR)

Following our published procedures [10,33,58,59], RNA was isolated from liver and subcutaneous adipose tissue. RT-qPCR was performed for *carnitine palmitoyltransferase 1 α* (*CPT1 α)*, *lipoprotein lipase* (*LPL*), *cluster of differentiation 36 molecule* (*CD36*), *fatty acid synthase* (*FAS*), *acetyl-CoA carboxylase* (*ACC*), *hormone-sensitive lipase* (*HSL*), *hydroxyacyl-CoA dehydrogenase* (*HADH*), *sterol regulatory element binding transcription factor 1* (*SREBP-1*), *peroxisome proliferator activated receptor alpha* (*PPARα*), and *PPARG coactivator 1 alpha* (*PGC1α*). The primer sequences were obtained from other studies [60,61,62,63,64,65,66]. Details on primers used are listed in Table S1. The relative abundances of target gene transcripts were calculated using Ct values for target and housekeeping genes using the 2^−∆∆CT^ method.

### 2.10. Immunoblot Analysis

Western blots were performed in the liver and kidney for *carbamoyl phosphate synthetase I (CPS I)*, *ornithine transcarbamylase (OTC)*, *argininosuccinate synthase 1 (ASS 1)*, *arginase 1 (ARG 1)*, and *argininosuccinate lyase (ASL)*, as we previously described [10,33,67]. *β-actin* and *GAPDH* were employed as loading controls to establish the relative amount of target protein abundance. The details on the antibodies used are given in Table S2.

### 2.11. Statistical Analysis

Overall growth, cumulative hourly FI, thermal radiation, and all other data obtained from laboratory analyses, including plasma metabolites, RT-qPCR, and western blot data were analyzed by a general linear model (GLM) procedure with the Tukey post-hoc following an outlier test, which was used based on the Interquartile Rule in SPSS (IBM SPSS Statistics Version 23, Armonk, NY, USA). The hourly, daily, and weekly recorded data, including FI, WI, BW, BWG, MFI, CFI, CPI, G:F, and G:P, were subjected to a mixed analysis with the fixed effects of diet, time, and interaction of diet by time and a random variable of animal in the model. The statistical model used for mixed analysis is defined as: *y_ijt_*  =  μ  +  α*_i_*  +  *d_j(i)_* + γ*_t_* + (αγ)*_it_* + *e_ijt_*, where *y_iij_* is the observation measured at time *t* on the *j*th pig assigned to the *i*th diet, μ is the overall mean effect, α_i_ is the *i*th fixed diet effect, *d_j(i)_* is the random effect of the *j*th pig within the *i*th diet, γ*_t_* is the fixed *t*th time effect when the measurement was taken, (αγ)*_it_* is the fixed interaction effect between diet and time, and *e_ijt_* is the random error associated with the *j*th pig assigned to the *i*th diet at time *t*. The lowest quantities of the fit statistics for corrected Akaike Information Criterion and Bayesian Information Criterion were used to model covariance structure for repeated measurements for each variable. For plasma glucose after a meal test, a paired Student’s *t*-test followed by a Benjamini–Hochberg correction with a 0.1 false discovery rate was used to determine the differences between the means of five preplanned comparisons: PC 0 min vs. PC 120 min, NC 0 min vs. NC 120 min, HV 0 min vs. HV 120 min, HI 0 min vs. HI 120 min, and HVI 0 min vs. HVI 120 min. The area under the curve (AUC) for cholesterol was determined by the Trapezoid rule, which sums the areas of all the trapezoids that were created between two time points, using the following equation: *AUC*_(*ti* −_ *_ti_*_−1)_ = (*t_i_* − *t_i_*_−1_) $\times \frac{f\left(ti\right)+f\left(ti-1\right)}{2}$ [68], where *f*$\left(ti\right)$ and *f*$\left(ti-1\right)$ are concentrations of blood cholesterol measured at two consecutive time points (i.e., *t_i_* and *t_i_*_−1_). Differences among treatments were considered significant at *p* ≤ 0.05 and a trend at 0.05 < *p* ≤ 0.10.

## 3. Results

### 3.1. Growth Measurements

**Figure 1:**
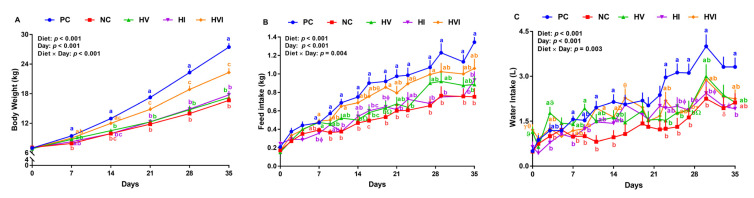
(**A**) Body weight, (**B**) feed intake, and (**C**) water intake of nursery pigs fed with very low protein diets containing isoleucine (Ile), valine (Val), or mix of both above NRC levels. PC: positive control, standard protein diet; NC: negative control, very low protein diet containing the first four limiting amino acids (i.e., lysine, methionine, threonine, and tryptophan) at NRC levels; HV: NC containing Val above NRC level; HI: NC containing Ile above NRC level; and HVI: NC containing both Val and Ile above NRC level. The values are means ± standard error of the mean. *n* = 8. ^a,b,c,ab^ the means with different superscript letter(s) at each time point are different (*p* ≤ 0.05). ^δ^ *p* ≤ 0.1 PC vs. NC, ^Ώ^ *p* ≤ 0.1 HV vs. PC, ^γ^ *p* ≤ 0.1 HVI vs. PC, ^ϕ^ *p* ≤ 0.1 HI vs. PC, ^ε^ *p* ≤ 0.1 HVI vs. HV, ^ϑ^ *p* ≤ 0.1 HV vs. NC, and ^θ^ *p* ≤ 0.1 HVI vs. NC.

No differences in animals’ initial BW were seen among the groups (Table 3). Compared to PC, NC, HV, HI, and HVI had a lower final BW (39, 37, 35, and 19%, respectively). HVI had a higher final BW than NC, HV, and HI by 34, 30, and 26%, respectively. Relative to PC, NC, HV, and HI reduced the ADG by 50, 50, and 47%, respectively (Table 3). The ADG of pigs fed with HVI was lower (24%) than the PC, and was 52, 52, and 42% higher than NC, HV, and HI, respectively. The effect of diet on BWG was significant throughout all weeks (Table S3). Relative to PC, NC reduced the BW by 16–39% throughout the study (*p* < 0.05; Figure 1A). HV and HI had a lower BW than PC on days 14–35 (*p* < 0.01; Figure 1A). HVI and PC had a similar BW on days 7 and 14, but HVI had a 14–19% lower BW than PC in the last three weeks of study. While HI and HV had a similar BW as NC, HVI had a 21–37% higher BW than NC on days 14–35. Relative to HV and HI, HVI had a higher BW on days 14–35 (*p* < 0.01; Figure 1A). BW of HV remained unchanged when compared to HI in the entire study (Figure 1A).

Compared to PC, NC, HV, and HI reduced the ADFI by 38, 26, and 33%, respectively, but HVI showed no difference compared to PC (Table 3). Pigs fed with HV and HI had a similar ADFI as NC, while HVI had 47% higher ADFI than NC. Further, HVI had 34% higher ADFI than HI (Table 3). Compared with PC, pigs fed with NC, HV, HI, and HVI had a lower ADPI. While ADPI for HV and HI showed no difference compared to NC, pigs fed with HVI had 43% higher ADPI than NC. Further, HVI tended to have a higher ADPI than HI. The effect of diet on MFI, CFI, and CPI was significant in all weeks (Table S3). Compared to PC, pigs fed with NC reduced the FI on day 9 onward (33–46%) (*p* < 0.05; Figure 1B). HV had a lower or tended to have a lower FI than PC on days 14, 16, 19, 23, and 35, and HI either had a lower or tended to have a lower FI than PC on days 7, 11, 16, 19, 21, 27, 29, 33, and 35 (Figure 1B). Pigs fed with HVI had a similar FI compared to PC on most experimental days (Figure 1B). Relative to NC, FI of HV and HI showed no difference on day 7 onward (Figure 1B). HVI had a higher and tended to have a higher FI than NC on days 11, 16, 19, 23, and 27 (Figure 1B). The FI of HVI showed no difference relative to HV in the entire experiment except for day 19 (Figure 1B). Pigs fed with HI had a lower FI relative to HVI on days 7, 11, 19, and 27 (*p* < 0.05; Figure 1B). The effect of dietary treatments on hourly FI on some representative experimental days is shown in Figure S2.

ADWI was lower in NC, HV, and HI compared to PC (45, 26, 33, and 24%, respectively); however, HVI had a similar ADWI as PC (Table 3). WI was either lower or tended to be lower in pigs fed with NC compared to PC on days 7, 11, 14, 23, 25, 27, 30, and 33 (*p* < 0.05; Figure 1C). HV had a lower WI than PC on days 23, 25, and 27 (*p* < 0.05; Figure 1C). WI was either lower or tended to be lower in pigs fed with HI than PC on days 7, 25, 27, 30, 33, and 35 (*p* < 0.05; Figure 1C). HVI had a similar WI compared to PC except on day 27, when the WI tended to be lower for HVI (*p* < 0.05; Figure 1C).

Compared with PC, pigs fed with NC, HV, HI, and HVI had 19, 26, 19, and 18% lower G:F, respectively (*p* < 0.01; Table 3). No differences among groups were found when G:F of NC was compared to HV, HI, and HVI (*p* > 0.1; Table 3). HVI tended to have a higher G:F than HV. Compared with PC, the G:P ratio was higher in pigs fed with NC and HVI and tended to be higher in HI. Relative to NC, HI and HVI had a similar G:P, while HV had a lower G:P. HVI tended to have a higher G:P than HV (Table 3). There was no difference among treatments on W:F ratio (Table 3). Weekly G:F and G:P are shown in Table S3.

Compared to PC, body length was lower in NC, HV, and HI; however, there was no difference with HVI (Table 3). Relative to NC, HV and HI had a similar body length, while it was higher in HVI by 11%. Relative to HV, and HI, the body length of HVI group was higher by 7 and 11%, respectively. HV and HI had a similar body length. Heart girth was lower in NC, HV, HI, and HVI than PC by 17, 17, 14, and 8%, respectively (Table 3). Relative to NC, no differences in heart girth were detected for HV and HI, but HVI had higher heart girth by 11%. Further, heart girth of HVI was 11% higher than HV. While relative to PC, wither height was lower in pigs fed with NC, HV, and HI (14, 16, and 14%, respectively), no differences in wither height were seen between HVI and PC (Table 3). Compared to NC, HV, HI, and HVI showed no difference, but HVI had a higher wither height than HV.

### 3.2. Thermal Radiation

**Figure 2:**
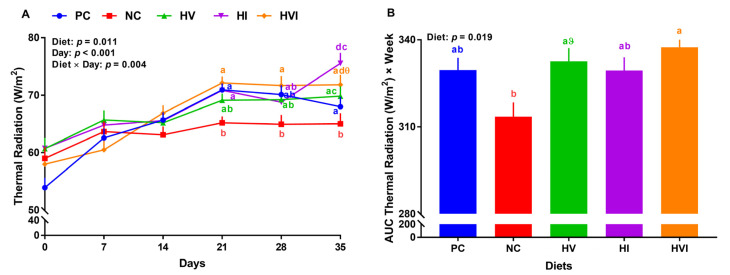
(**A**) Thermal radiation and (**B**) area under the curve (AUC) for thermal radiation in nursery pigs fed with very low protein diets containing isoleucine (Ile), valine (Val), or mix of both above NRC levels. PC: positive control, standard protein diet; NC: negative control, very low protein diet containing the first four limiting amino acids (i.e., lysine, methionine, threonine, and tryptophan) at NRC levels; HV: NC containing Val above NRC level; HI: NC containing Ile above NRC level; and HVI: NC containing both Val and Ile above NRC level. The values are means ± standard error of the mean. *n* = 8. ^a,b,ab^ Among groups, the means with different superscript letter(s) at each time point are different (*p* ≤ 0.05). ^ϑ^ *p* ≤ 0.1 HV vs. NC, ^θ^ *p* ≤ 0.1 HVI vs. NC.

Overall, the effects of diet, day, and diet × day on thermal radiation were significant (*p* < 0.05; Figure 2A). No significant differences in thermal radiation of different groups were detected during the first two weeks. Relative to PC, NC reduced thermal radiation by 12, 8, and 4%, on weeks 3 to 5, respectively (Figure 2A). Thermal radiation of HV, HI, and HVI showed no difference compared to PC throughout the study, except for HI that had a higher thermal radiation on day 35. Thermal radiation of HI was higher than NC on weeks 3 and 5 by 9 and 16%, respectively (*p* < 0.01; Figure 2A). Relative to NC, HVI had a higher thermal radiation of 10% on weeks 3 to 5. Relative to PC, thermal radiation of dietary treatments did not change (Figure 2B). Compared to NC, the AUC thermal radiation of HVI was higher by 8% (*p* < 0.01; Figure 2B). Furthermore, the AUC thermal radiation tended to increase in HV in comparison with NC. No differences in AUC thermal radiation were detected when HVI, HI, and HVI groups were compared.

### 3.3. Plasma Glucose, Triglycerides, Cholesterol, and BUN

**Figure 3:**
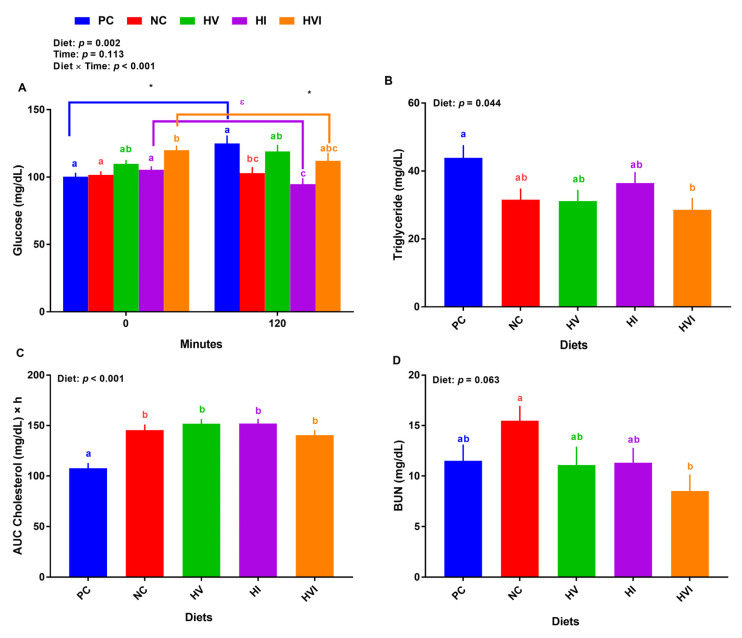
Plasma metabolite of nursery pigs fed with very low protein diets containing isoleucine (Ile), valine (Val), or mix of both above NRC levels. (**A**) glucose concentration at baseline (0 min) and 120 min after meal, (**B**) triglyceride concentration, (**C**) area under the curve (AUC) for cholesterol, and (**D**) blood urea nitrogen (BUN). PC: positive control, standard protein diet; NC: negative control, very low protein diet containing the first four limiting amino acids (i.e., lysine, methionine, threonine, and tryptophan) at NRC levels; HV: NC containing Val above NRC level; HI: NC containing Ile above NRC level; and HVI: NC containing both Val and Ile above NRC level. The values are means ± standard error of the mean. *n* = 8. ^a,b,c,ab,bc,abc^ Among groups, the means with different superscript letter(s) are different (*p* ≤ 0.05). * *p* ≤ 0.05 0 min vs. 120 min; ^ε^ *p* ≤ 0.1 0 min vs. 120 min.

Overall, the effects of diet and diet × day on plasma glucose were significant (*p* < 0.01; Figure 3A). At baseline, the plasma glucose for pigs fed with NC, HI, and HV showed no difference relative to PC, but HVI had a higher plasma glucose than PC (Figure 3A). Compared to NC, HV and HI showed no difference while HVI had a higher plasma glucose. HVI had a higher plasma glucose than HI at baseline. At 2-h post meal, relative to PC, NC and HI had a lower plasma glucose (Figure 3A). Further, HI had a lower plasma glucose than HV. Comparing plasma glucose for baseline and post meal, PC had a greater glucose at 2-h post meal than the baseline. Plasma glucose of HVI and HI at 2-h post meal was lower and tended to be lower than baseline, respectively (Figure 3A). Compared to PC, HVI had a lower TG, but NC, HV, and HI showed no difference (Figure 3B). In comparison with PC, all groups increased the AUC cholesterol (Figure 3C). Relative to NC, HVI had a lower BUN (Figure 3D).

### 3.4. The mRNA Abundance of Key Regulatory Genes of Lipid Metabolism in Liver and Subcutaneous Adipose Tissue

**Figure 4:**
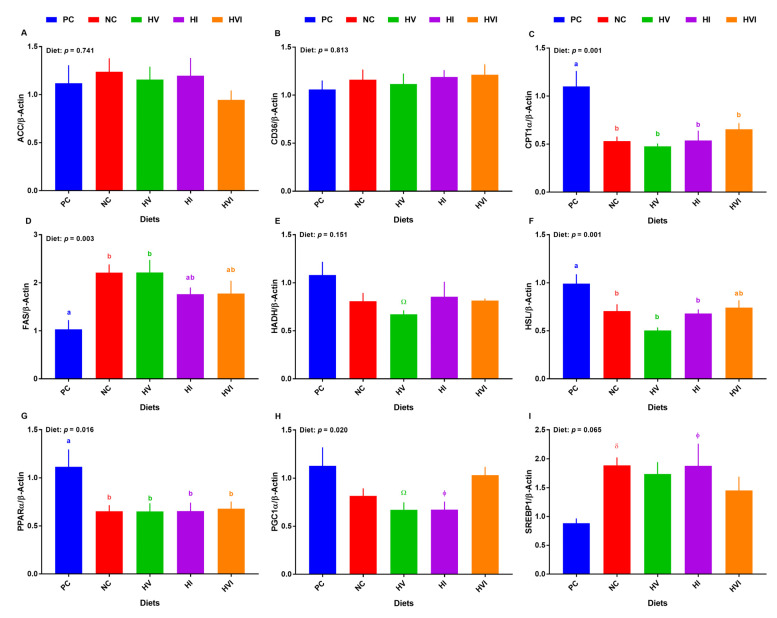
mRNA abundance of lipid metabolism markers in liver of nursery pigs fed with very low protein diets containing isoleucine (Ile), valine (Val), or mix of both above NRC levels. (**A**) *acetyl-CoA carboxylase alpha* (*ACC*), (**B**) *cluster of differentiation 36 molecule* (*CD36*), (**C**) *carnitine palmitoyltransferase I α* (*CPT1α*), (**D**) *fatty acid synthase* (*FAS*), (**E**) *hydroxyacyl-CoA dehydrogenase* (*HADH*), (**F**) *hormone-sensitive lipase* (*HSL*), (**G**) *peroxisome proliferator activated receptor alpha* (*PPARα*), (**H**) *PPARγ coactivator 1 alpha* (*PGC1α*), and (**I**) *sterol regulatory element-binding protein 1* (*SREBP-1*). PC: positive control, standard protein diet; NC: negative control, very low protein diet containing the first four limiting amino acids (i.e., lysine, methionine, threonine, and tryptophan) at NRC levels; HV: NC containing Val above NRC level; HI: NC containing Ile above NRC level; and HVI: NC containing both Val and Ile above NRC level. The values are means ± standard error of the mean. *n* = 8. ^a,b,ab^ Among groups, the means with different superscript letter(s) are different (*p* ≤ 0.05). ^δ^ *p* ≤ 0.1 PC vs. NC, ^Ώ^ *p* ≤ 0.1 HV vs. PC, ^ϕ^ *p* ≤ 0.1 HI vs. PC.

Overall, the effect of diet on the transcript of hepatic *CPT1α*, *FAS*, *HSL*, *PPARα*, and *PGC1α* was significant (Figure 4C,D,F–H), and on mRNA abundance of hepatic *SREBP-1* tended to be significant (Figure 4I). No differences among treatments were detected for the mRNA abundance of hepatic *ACC* and *CD36* (Figure 4A,B). The mRNA abundance of *CPT1α* was lower in all groups compared to PC, but no differences among NC, HV, HI, and HVI were detected (Figure 4C). The transcript abundance of *FAS* was greater in NC and HV relative to PC, but showed no difference for HI and HVI, when compared to PC (Figure 4D). The gene expression of *HADH* tended to be lower in HV compared to PC (Figure 4E). Relative to PC, the transcript of hepatic *HSL* was reduced in NC, HV, and HI, but not in HVI (Figure 4F). Compared to PC, the hepatic transcript of *PPARα* was lower in all dietary treatments (*p <* 0.05; Figure 4G). The transcript of *PPARα* showed no difference among NC, HV, HI, and HVI. Relative to PC, the mRNA abundance of *PGC1α* tended to be lower in HV and HI (*p <* 0.01; Figure 4H). The mRNA abundance of *SREBP-1* tended to be higher in NC and HI when compared with PC (*p <* 0.1; Figure 4I).

Overall, the effect of diet on the gene expression of *LPL* in subcutaneous adipose tissue was significant (Figure S3E). No differences were detected among treatments for the mRNA abundance of *ACC*, *CD36*, *FAS*, *HADH*, *PPARα*, *PGC1α*, and *SREBP-1* in subcutaneous adipose tissue (Figure S3A–D,F–H). Relative to PC, the transcript of *LPL* in adipose tissue was higher in NC and tended to be higher in HV, but not in HI and HVI (Figure S3E). The mRNA abundance of *LPL* tended to be lower in HI and HVI compared to NC and HV, respectively (Figure S3E).

### 3.5. The Protein Abundance of Key Regulatory Enzymes of Urea Cycl in the Liver and Kidney

**Figure 5:**
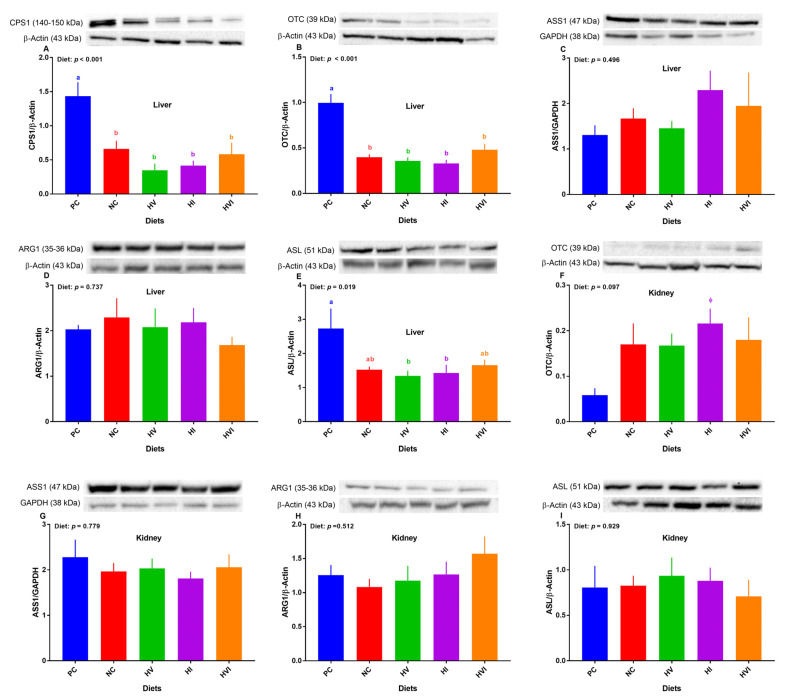
Relative protein abundance of urea cycle enzymes in the liver (**A**–**E**) and kidney (**F**–**I**) of nursery pigs fed with very low protein diets containing isoleucine (Ile), valine (Val), or mix of both above NRC levels. (**A**) *carbamoyl phosphate synthetase I (CPS1*), (**B**,**F**) *ornithine transcarbamylase (OTC)*, (**C**,**G**) *argininosuccinate synthase I (ASS1)*, (**D**,**H**) *arginase I (ARG1)*, and (**E**,**I**) *argininosuccinate lyase (ASL). GAPDH* and *β-actin* were selected as a loading control. PC: positive control, standard protein diet; NC: negative control, very low protein diet containing the first four limiting amino acids (i.e., lysine, methionine, threonine, and tryptophan) at NRC levels; HV: NC containing Val above NRC level; HI: NC containing Ile above NRC level; and HVI: NC containing both Val and Ile above NRC level. The values are means ± standard error of the mean. ^a,b,ab^ Among groups, the means with different superscript letter(s) are different (*p* ≤ 0.05). *n* = 8. ^ϕ^ *p* ≤ 0.1 HI vs. PC.

Overall, the effect of diet on protein expression of hepatic *CPS-1α*, *OTC*, and *ASL* was significant (Figure 5A,B,E). Relative to PC, the protein abundance of *CPS-1α* and *OTC* were lower in all dietary treatments (*p* < 0.01; Figure 5A,B), but showed no difference among NC, HV, HI, and HVI. No differences among treatments were detected for the protein abundance of *ASS1* and *ARG1* (Figure 5C,D). Compared to PC, NC and HVI showed no difference in protein abundance of *ASL*, but did show a decrease in HV and HI (Figure 5E). The protein abundance of *ASL* showed no difference among NC, HV, HI, and HVI (Figure 5E).

Overall, the effect of diet on the protein expression of *OTC* in the kidney tended to be significant (*p* < 0.1; Figure 5F). In comparison with PC, the protein abundance of *OTC* tended to increase in HI (Figure 5F). No differences among treatments were detected for the protein abundance of *ASS1*, *ARG1*, and *ASL* in the kidney (Figure 5G–I).

## 4. Discussion

Supplementation of BCAAs or mixture of Ile and Val not only decrease the negative impact of VLP diets on the growth of pigs, but also reduce BUN, and influence lipid metabolism [9,10,11,33]. Little is understood on the mechanisms by which BCAAs regulate the N utilization and lipid metabolism in pigs. The objective of the current study was to assess the effect of dietary Ile and Val combination on gene or protein expression of key rate limiting enzymes involved in ureagenesis and fat metabolism (lipolysis and lipogenesis) in target tissues of pigs offered with VLP diets. Here we showed that: 1) HVI partially improved the growth and completely recovered the FI of pigs fed with VLP diets; 2) pigs fed with NC and HV seem to promote lipogenesis, but HVI normalizes the increased rate of lipogenesis; and 3) all low protein groups (i.e., NC, HV, HI, and HVI) reduced the protein expression of rate-limiting enzymes of ureagenesis in the liver. Overall, a combination of dietary Val and Ile improved the growth and FI and decreased the rate of lipogenesis induced by low protein diets.

Feeding pigs with a VLP diet supplemented with the first four limiting AAs decreased the BW and ADG, which is consistent with previous research [5,6,7,8]. In line with other studies [19,69,70], adding a combination of Val and Ile above NRC levels improved the growth and FI of pigs fed with VLP diets. In particular, HVI completely recovered the FI of VLP group to the levels seen in PC pigs. This is in parallel with our recent data showing that supplementation of a combination of Val and Ile at NRC level [11] or Val above and Ile at NRC levels [53] recovers the FI of pigs fed with VLP diets. Improved FI in HVI group could be explained by changes in expression of peptides involved in FI regulation. Previously, we and others showed that the transcript of *orexigenic neuropeptide Y* and *agouti-related protein* was increased, and that for *anorexigenic proopiomelanocortin*, *melanocortin-4-receptor*, and *cocaine- and amphetamine regulated transcript* was decreased in the hypothalamus when BCAAs or Val were added to low protein diets [14,33,71,72].

While HVI completely recovered the FI, it showed only a partial improvement in BW and ADG. The lack of complete recovery in the growth of the HVI group might be attributed to a greater thermal radiation (i.e., energy loss) in this group, which is consistent with our previous data [11]. The higher thermal radiation in HVI group is likely due to AA imbalances caused by a higher ratio of dietary Val and Ile to Lys and other AAs. The AA imbalances in HVI group may not only influence the growth rate through reducing the availability of energy for wight gain, but also may alter the efficiency of nutrients utilization [73]. The mechanisms by which the AA imbalances are sensed and possibly increase the energy expenditure [74] are not fully elucidated and further research is required. The first step of BCAAs degradation is catalyzed by a common enzyme, branched-chain α-ketoacid dehydrogenase, and BCAAs share the same transport system with other AAs. Whether shared catabolic pathways and transport systems among BCAAs and other AAs reduce the supply of Leu and essential AAs in HVI group and whether that contributes to a higher thermal radiation in this group remains to be determined.

HVI pigs appeared to have a better glucose clearance than those fed with standard protein diets. Similarly, others showed that individual intrahypothalamic infusion of Ile and Val in rats reduced postprandial blood glucose by lowering the hepatic glucose production [75]. In the current study, HI improved the glucose clearance after the meal test. This was in line with previous studies showing that intragastric infusion of Ile and Leu in healthy males [76,77] or oral administration of Ile in rats [78,79,80] and healthy humans [81], but not Val, decreased the postprandial blood glucose. This is while intravenous infusion of Val in healthy humans [82] or its oral administration in rats [79] either marginally decreased or raised blood glucose concentration, respectively. Therefore, in our study the improved glucose tolerance in the HVI group seems to be linked with the role of Ile in reducing blood glucose concentrations. The hypoglycemic effects of Ile have been attributed to reduced hepatic gluconeogenesis and increased glucose uptake in skeletal muscle [78,80].

Little is understood on the role of Ile and Val on lipid metabolism in pigs under protein restriction. Here we showed that NC and HV induced lipogenesis through increasing the gene expressions of *FAS* in the liver and *LPL* in adipose tissue and reducing transcript of hepatic *HSL*. All low protein groups (i.e., NC, HV, HI, and HVI) had a lower abundance *CPT1α* in the liver than that in the PC group. Similar to our data, others showed that low protein diets reduce the hepatic expression of *CPT1α* in laying hens [83], but high protein diets increase hepatic mRNA abundance of *CPT1α* in mice [84]. Further, here we showed that all low protein groups increased the blood cholesterol, which is in line with our and other previous studies showing that by reducing the dietary protein content, plasma cholesterol increases in pigs [7,85]. In the present study, HVI seems to mitigate the rate of lipogenesis through normalizing the transcript of hepatic *FAS*, *SREBP1*, *HSL* and *PGC1α*, and *LPL* in adipose tissue. HVI also reduced the blood TG concentration. Likewise, we and others previously showed that BCAAs supplementation decreases the blood TG concentration in pigs and obese rats [33,86]. The inhibitory role of HVI on lipogenesis could be due to contradictory effects of Ile and Val on fat metabolism. While HV seems to have more positive effects on lipogenesis via decreasing the transcript of hepatic *HADH*, *HSL*, and *PGC1α*, HI appears to reduce the lipogenesis by normalizing the hepatic *FAS* and *LPL* in adipose tissue. Ile has been previously shown to stimulate lipolysis and reduce fat deposition, white adipose tissue mass, and TG concentration in the muscle and liver of mice [35]. Supplementation of Ile in drinking water of obese mice [36] and in the diet of broilers [87] has shown to reduce the white adipose tissue mass and body weight and the serum TG, but Val supplementation increased serum TG in mice fed with high-fat diet [38]. Therefore, the protective effects of HVI on lipogenesis might be explained by the buffering effect of Ile on Val-induced lipogenesis. Others showed that BCAAs-supplemented mice had less weight, adipose tissue, and hepatic TG content and downregulated the lipogenic enzymes in liver compared to their control counterparts [34,37].

Branched-chain AAs improve N utilization possibly through multiple mechanisms including increasing the activity and/or secretion rate of proteolytic enzymes [23,24,25,26] and expression of AAs transporters in gut [27,28,29] and providing N for endogenous AAs synthesis [30,31,32]. Little information is available on whether BCAAs, and in particular Ile and Val alone or in combination, affect the N balance through regulation of ureagenesis in the liver and kidney. HVI had a lower BUN than NC in the present study. This is in parallel with previous studies showing that VLP diets supplemented with BCAAs [9,10] and a mixture of Ile and Val [11], or standard protein diets supplemented with Val [12,13,14] and Ile [15,88] had less BUN than un-supplemented pigs. A lower BUN in HVI group is suggestive of an improved N retention [16,17]. Previous studies have indicated that BCAAs improve the efficiency of AAs and N utilization in pigs [18,19,89], and humans [20,21,22]. Although HVI had a lower BUN than NC, there were no differences in protein abundance of the rate limiting enzymes of the urea cycle in the liver and kidney between these two groups. Whether a higher BUN of NC versus HVI downregulates the urea cycle enzymes through a negative feedback mechanism, as previously suggested [90], remains to be determined. Further, all low protein groups (i.e., NC, HV, HI, and HVI) decreased the ureagenesis through the suppression of the protein expression of rate-limiting enzymes of ureagenesis, *CPS1, OTC*, and *ASL*, in the liver. The caveat of our study is that we measured the BUN at a single time point at the end of study and it is not clear whether BUN concentration follows the same pattern in early stages of dietary interventions, and whether those chronic changes have regulatory effects on urea cycle enzymes expression.

## 5. Conclusions

Supplementation of a mixture of dietary Val and Ile partially improved the growth and fully recovered the FI of pigs fed with VLP diets. A partial, but not complete recovery of growth, following supplementation of a mixture of Ile and Val to VLP diets, is likely due to increased energy loss. Pigs fed with VLP diets w/o Val supplementation enhanced lipogenesis by increasing the mRNA abundance of hepatic *FAS* and adipose *LPL*, and reducing transcript of hepatic *HSL*. A combination of Val and Ile added to VLP diets improved the glucose tolerance and reduced the rate of lipogenesis induced by protein deficient diets through normalizing the transcript of hepatic *FAS*, *SREBP1*, *HSL* and *PGC1α*, and *LPL* in adipose tissue. All low protein groups (i.e., NC, HV, HI, and HVI) suppressed the protein abundance of rate-limiting enzymes of ureagenesis, *CPS1*, *OTC* and *ASL*, in the liver. Further research is required to explore the regulatory role of BUN on ureagenesis under acute and chronic treatments with Ile and Val.

## Figures and Tables

**Table 1:** Ingredients and chemical composition of experimental diets (as-fed basis).

	Diets ^1^												
	N1		N2						N3				
Ingredients ^2^, %			PC	NC	HV	HI	HVI		PC	NC	HV	HI	HVI
Corn, yellow dent	37.41		55.60	75.54	75.41	75.40	75.27		69.16	87.14	87.02	87.01	86.89
Soybean meal, 47.5% CP	18.00		21.67	2.42	2.42	2.42	2.42		18.60	1.10	1.10	1.10	1.10
Fish meal, menhaden	6.00		4.29	4.29	4.29	4.29	4.29		4.29	4.29	4.29	4.29	4.29
Whey, dried	24.10		3.00	3.00	3.00	3.00	3.00		-	-	-	-	-
Corn starch	-		9.43	6.92	6.92	6.92	6.92		3.25	1.16	1.16	1.16	1.16
Lactose	6.80		-	-	-	-	-		-	-	-	-	-
Plasma spray-dried	5.29		3.10	3.10	3.10	3.10	3.10		2.10	2.00	2.00	2.00	2.00
Corn oil	0.49		-	-	-	-	-		-	-	-	-	-
Dicalcium phosphate 18.5%	0.86		1.25	1.63	1.63	1.63	1.63		1.05	1.41	1.41	1.41	1.41
Limestone	0.38		0.48	0.36	0.36	0.36	0.36		0.40	0.31	0.31	0.31	0.31
Salt	0.16		0.52	0.52	0.52	0.52	0.52		0.47	0.47	0.47	0.47	0.47
Vitamin premix	0.04		0.05	0.05	0.05	0.05	0.05		0.05	0.05	0.05	0.05	0.05
Trace mineral premix	-		0.02	0.02	0.02	0.02	0.02		0.01	0.01	0.01	0.01	0.01
Zinc oxide, 72% Zn	0.01		0.01	0.01	0.01	0.01	0.01		0.01	0.01	0.01	0.01	0.01
L-Lysine, HCl	0.27		0.36	0.85	0.85	0.85	0.85		0.38	0.84	0.84	0.84	0.84
DL-methionine	0.13		0.11	0.19	0.19	0.19	0.19		0.09	0.16	0.16	0.16	0.16
L-threonine	0.05		0.10	0.36	0.36	0.36	0.36		0.12	0.36	0.36	0.36	0.36
L-tryptophan	0.01		0.01	0.11	0.11	0.11	0.11		0.02	0.11	0.11	0.11	0.11
L-isoleucine	-		-	-	-	0.42	0.42		-	-	-	0.39	0.39
L-valine	-		-	-	0.48	-	0.48		-	-	0.44	-	0.44
L-alanine	-		-	0.63	0.28	0.35	-		-	0.58	0.26	0.32	-
**Calculated Chemical Composition** ^3^													
Dry matter, %	90.32		90.00	89.63	89.65	89.65	89.67		88.81	88.70	88.74	88.74	88.76
ME, Mcal/kg	3.40		3.40	3.40	3.40	3.40	3.40		3.35	3.35	3.35	3.35	3.35
Crude protein, %	22.00		20.08	14.00	14.00	14.00	14.00		18.60	13.00	13.00	13.00	13.00
Crude fiber, %	1.67		2.25	1.86	1.86	1.86	1.86		2.42	2.07	2.06	2.06	2.06
Crude fat, %	3.36		3.07	3.40	3.39	3.39	3.39		3.44	3.72	3.72	3.72	3.71
Calcium, %	0.85		0.80	0.80	0.80	0.80	0.80		0.70	0.70	0.70	0.70	0.70
Total phosphorus, %	0.70		0.65	0.65	0.65	0.65	0.65		0.60	0.60	0.60	0.60	0.60
Available phosphorus, %	0.61		0.47	0.52	0.52	0.52	0.52		0.40	0.45	0.45	0.45	0.45
SID Lysine, %	1.50		1.35	1.35	1.35	1.35	1.35		1.23	1.23	1.23	1.23	1.23
SID Threonine, %	0.88		0.79	0.79	0.79	0.79	0.79		0.73	0.73	0.73	0.73	0.73
SID Methionine, %	0.43		0.39	0.39	0.39	0.39	0.39		0.36	0.36	0.36	0.36	0.36
SID Tryptophan, %	0.25		0.22	0.22	0.22	0.22	0.22		0.20	0.20	0.20	0.20	0.20
SID Isoleucine, %	0.79		0.71	0.39	0.39	0.81	0.81		0.64	0.35	0.35	0.74	0.74
SID Valine, %	0.96		0.86	0.53	1.01	0.53	1.01		0.78	0.48	0.92	0.48	0.92
SID Leucine, %	1.65		1.52	1.08	1.08	1.08	1.08		1.44	1.03	1.03	1.03	1.03
SID Histidine, %	0.50		0.47	0.30	0.30	0.30	0.30		0.44	0.28	0.28	0.28	0.28
SID Arginine, %	1.14		1.14	0.59	0.59	0.59	0.59		1.04	0.54	0.54	0.54	0.53
SID Phenylalanine, %	0.90		0.85	0.50	0.50	0.50	0.50		0.78	0.46	0.46	0.46	0.46
SID Valine: SID Lysine	0.64		0.64	0.39	0.75	0.39	0.75		0.63	0.39	0.75	0.39	0.75
SID Isoleucine: SID Lysine	0.52		0.53	0.29	0.29	0.60	0.60		0.52	0.28	0.28	0.60	0.60

^1^ Diets were formulated using National Swine Nutrition Guide (NSNG; Version 2.1 Metric, ^©^2012 U.S. Pork Center of Excellence). PC: positive control, standard protein diet; NC: negative control, very low protein diet containing the first four limiting amino acids (i.e., lysine, methionine, threonine, and tryptophan) at NRC levels; HV: NC containing valine (Val) above NRC level; HI: NC containing isoleucine (Ile) above NRC level; and HVI: NC containing both Val and Ile above NRC level. N1 (nursery phase 1): this diet was provided from day 1 to 7 of the study; N2 (nursery phase 2): these diets were fed from day 8 to 21 of the study; and N3 (nursery phase 3): these diets were offered from day 22 to 42 of the study. ^2^ Corn, soybean meal, fish meal, whey, corn starch, lactose, plasma spray-dried, corn oil, dicalcium phosphate, limestone, salt, zinc oxide, vitamin premix, trace mineral premix, DL-methionine (99%), and L-lysine HCl (79–99%) were purchased by Nutra Blend, LLC (Neosho, MO, USA). L-threonine (98.5%) and L-tryptophan (98%) were purchased from Ajinomoto (Overland Park, KS, USA). L-isoleucine (98.5%), L-alanine, and L-valine (96.5%) were provided from Ajinomoto Health & Nutrition North America, Inc. (Raleigh, NC, USA). Vitamin premix contained: vitamin A, 1,653,750 IU/kg; vitamin D3, 661,500 IU/kg; vitamin E, 17,640 IU/kg; vitamin K (menadione), 1323 mg/kg; vitamin B12, 13.23 mg/kg; niacin, 19,845 mg/kg; D-pantothenic acid, 11,025 mg/kg; riboflavin, 3307.5 mg/kg; and phytase, 300,056.4 FYT/kg. Trace mineral premix contained: copper, 11,000 ppm; iodine, 198 ppm; iron, 73,000 ppm; manganese, 22,000 ppm; selenium, 198 ppm; and zinc, 73,000 ppm. ^3^ ME: metabolize energy; SID: standard ileal digestibility.

**Table 2:** Analyzed chemical composition of experimental diets (as-fed basis).

	Diets ^1^												
	N1		N2						N3				
Chemical Composition			PC	NC	HV	HI	HVI		PC	NC	HV	HI	HVI
Dry matter, %	91.30		89.30	89.10	89.50	89.10	89.30		88.80	88.30	88.70	88.50	88.70
Crude protein, %	20.40		19.50	13.50	13.60	14.00	13.80		19.30	12.30	12.70	13.80	12.60
Crude fiber, %	1.40		2.10	1.50	1.50	1.80	1.70		2.00	1.80	2.10	2.30	2.30
Calcium, %	0.95		0.81	0.77	0.77	0.80	0.75		0.83	0.71	0.85	0.80	0.80
Phosphorus, %	0.87		0.72	0.68	0.69	0.77	0.68		0.72	0.61	0.67	0.68	0.73
Taurine ^2^, %	0.19		0.19	0.19	0.19	0.19	0.21		0.19	0.20	0.21	0.19	0.20
Hydroxyproline, %	0.18		0.12	0.10	0.10	0.11	0.00		0.15	0.11	0.00	0.12	0.12
Aspartic acid, %	2.16		1.96	1.05	1.03	1.05	1.05		1.76	0.91	0.90	0.96	0.84
Threonine, %	1.09		0.90	0.84	0.92	0.95	0.91		0.85	0.77	0.87	0.76	0.70
Serine, %	0.96		0.88	0.54	0.54	0.55	0.61		0.80	0.48	0.51	0.51	0.47
Glutamic acid, %	3.45		3.37	1.96	1.93	1.97	2.02		3.04	1.79	1.83	1.92	1.73
Proline, %	1.28		1.22	0.86	0.83	0.86	0.82		1.14	0.80	0.81	0.84	0.78
Lanthionine ^2^, %	0.07		0.04	0.03	0.04	0.03	0.00		0.05	0.03	0.00	0.04	0.03
Glycine, %	0.96		0.91	0.59	0.59	0.60	0.61		0.85	0.55	0.54	0.56	0.52
Alanine, %	1.12		1.03	1.37	1.04	1.08	0.77		0.99	1.41	0.99	1.10	0.69
Cysteine, %	0.46		0.39	0.26	0.25	0.26	0.26		0.34	0.23	0.22	0.24	0.20
Valine, %	1.16		1.01	0.64	1.11	0.65	1.14		0.90	0.56	0.97	0.59	0.91
Methionine, %	0.52		0.48	0.38	0.43	0.38	0.40		0.42	0.37	0.39	0.33	0.31
Isoleucine, %	0.92		0.83	0.47	0.45	0.86	0.93		0.74	0.41	0.41	0.80	0.74
Leucine, %	1.82		1.67	1.15	1.13	1.16	1.17		1.56	1.06	1.07	1.12	1.04
Tyrosine, %	0.72		0.65	0.35	0.36	0.37	0.40		0.59	0.33	0.33	0.32	0.33
Phenylalanine, %	0.97		0.95	0.57	0.56	0.57	0.57		0.87	0.51	0.50	0.53	0.49
Hydroxylysine, %	0.01		0.01	0.01	0.01	0.01	0.01		0.02	0.01	0.01	0.01	0.01
Ornithine ^2^, %	0.02		0.02	0.01	0.01	0.01	0.01		0.02	0.01	0.01	0.01	0.01
Lysine, %	1.71		1.53	1.38	1.60	1.47	1.47		1.40	1.33	1.31	1.30	1.28
Histidine, %	0.54		0.51	0.32	0.32	0.32	0.33		0.47	0.29	0.29	0.31	0.28
Arginine, %	1.19		1.20	0.64	0.63	0.64	0.67		1.09	0.57	0.57	0.60	0.54
Tryptophan, %	0.29		0.27	0.22	0.23	0.24	0.21		0.23	0.19	0.20	0.20	0.19
Valine: Lysine	0.68		0.66	0.46	0.69	0.44	0.78		0.64	0.42	0.74	0.45	0.71
Isoleucine: Lysine	0.54		0.54	0.34	0.28	0.59	0.63		0.53	0.31	0.31	0.62	0.58

^1^ PC: positive control, standard protein diet; NC: negative control, very low protein diet containing the first four limiting amino acids (i.e., lysine, methionine, threonine, and tryptophan) at NRC levels; HV: NC containing valine (Val) above NRC level; HI: NC containing isoleucine (Ile) above NRC level; and HVI: NC containing both Val and Ile above NRC level. N1 (nursery phase 1): this diet was provided from day 1 to 7 of the study; N2 (nursery phase 2): these diets were fed from day 8 to 21 of the study; and N3 (nursery phase 3): these diets were offered from day 22 to 42 of the study. ^2^ Non-proteinogenic amino acids.

**Table 3:** Growth performance of nursery pigs fed with very low-protein diets containing isoleucine, valine or mix of both above NRC levels.

Measurements ^2^	Diets ^1^					SEM ^3^ *p*-Value	
	PC	NC	HV	HI	HVI		
Initial BW, kg	6.99	7.10	6.86	7.00	6.96	0.13	0.99
Final BW, kg	27.45 ^a^	16.64 ^b^	17.21 ^b^	17.76 ^b^	22.31 ^c^	0.78	<0.01
ADG, kg/day	0.58 ^a^	0.29 ^b^	0.29 ^b^	0.31 ^b^	0.44 ^c^	0.02	<0.01
ADFI, kg/day	0.86 ^a^	0.53 ^b^	0.64 ^bc^	0.58 ^b^	0.78 ^ac^	0.03	<0.01
ADPI, kg/day	0.17 ^a^	0.07 ^b^	0.08 ^bc^	0.08 ^b^	0.10 ^cτ^	0.01	<0.01
ADWI, L/day	2.36 ^a^	1.31 ^b^	1.74 ^b^	1.58 ^b^	1.80 ^ab^	0.08	<0.01
G:F, kg/kg	0.68 ^a^	0.55 ^bc^	0.50 ^b^	0.55 ^bc^	0.56 ^cε^	0.01	<0.01
G:P, kg/kg	3.54 ^a^	4.37 ^b^	3.83 ^ac^	3.98 ^bcϕ^	4.31 ^bε^	0.07	<0.01
W:F, L/kg	2.77	2.40	2.72	2.73	2.40	0.07	0.26
Final body length, m	0.64 ^a^	0.55 ^b^	0.57 ^b^	0.55 ^b^	0.61 ^a^	0.01	<0.01
Final heart girth, m	0.66 ^a^	0.55 ^b^	0.55 ^b^	0.57 ^bc^	0.61 ^c^	0.01	<0.01
Final wither height, m	0.44 ^a^	0.38 ^bc^	0.37 ^c^	0.38 ^bc^	0.41 ^aτ^	0.00	<0.01

^1^ PC: positive control, standard protein diet; NC: negative control, very low protein diet containing the first four limiting amino acids (i.e., lysine, methionine, threonine, and tryptophan) at NRC levels; HV: NC containing valine (Val) above NRC level; HI: NC containing isoleucine (Ile) above NRC level; and HVI: NC containing both Val and Ile above NRC level. The values are means, *n* = 8. ^2^ BW: body weight; ADG: average daily gain; ADFI: average daily feed intake; ADPI: average daily protein intake; ADWI: average daily water intake; G:F: gain:feed ratio; G:P: gain:protein ratio; and W:F: water:feed ratio. ^3^ SEM: standard error of the mean. ^a,b,c^ Within each row, the values with different superscript letter(s) are different (*p* ≤ 0.05). ^τ^ *p* ≤ 0.1 HVI vs. HI, ^ε^ *p* ≤ 0.1 HVI vs. HV, and ^ϕ^ *p* ≤ 0.1 HI vs. PC.

## Data Availability

Data is contained within the article or supplementary material.

## Supplementary Materials

The following supporting information can be downloaded at: https://www.mdpi.com/article/10.3390/metabo13010089/s1, Figure S1: Representative thermal images of dietary group; Figure S2: Feed intake of nursery pigs fed with very low protein diets containing Ile, Val or mix of both above NRC levels; Figure S3: mRNA abundance of lipid metabolism markers in subcutaneous adipose tissue of nursery pigs fed with very low protein diets containing Ile, Val or mix of both above NRC levels; Table S1: the sequences, location on template, amplicon size, and GenBank accession numbers for primers used for RT-qPCR; Table S2: the host, dilution, and vendor of antibodies for immunoblotting; Table S3: weekly growth performance of nursery pigs fed with very low-protein diets containing Ile, Val or mix of both above NRC levels.
