# Supplementary material for: Dietary Isoleucine and Valine: Effects on Lipid Metabolism and Ureagenesis in Pigs Fed with Protein Restricted Diets
Source: Metabolites. 2023 Jan 5;13(1):89. doi: 10.3390/metabo13010089 (PMC9861042; doi:10.3390/metabo13010089)
Supplement: Supplementary file 1 [file metabolites-13-00089-s001.zip › metabolites-2089730-supplementary.pdf]

Supplementary Material

Supplementary Tables

Table S1. The sequences [forward (F) and reveres (R)], location on template, amplicon size (bp), and GenBank accession numbers for primers used for reverse transcription quantitative real-time polymerase chain reaction (RT-qPCR)

**Genes[1]**	**Sequence (5' → 3')**	**Location on template**	**Amplicon length (bp)**	**GenBank accession no.**	**Reference**
FAS	**F:** CTGCTGAAGCCTAACTCCTCG<br>**R:** TTGCTCCTTGGAACCGTCTG	584 - 604<br>771 - 790	207	NM_001099930.1	[62]
ACC	**F:** ATGTTTCGGCAGTCCCTGAT<br>**R:** TGTGGACCAGCTGACCTTGA	4870 - 4889<br>4983 - 5002	133	NM_001114269.1	[62]
HSL	**F:** GCTCCCATCGTCAAGAATC<br>**R:** TAAAGCGAATGCGGTCC	2043 - 2061<br>2291 - 2307	265	NM_214315.3	[62]
PPARα	**F:** CATCCTCGCGGGAAAGG<br>**R:** GGCCATACACAGTGTCTCCATGT	722 - 738<br>769 - 791	70	NM_001044526.1	[65]
SREBP-1	**F**: CGGACGGCTCACAATGC<br>**R**: GACGGCGGATTTATTCAGCTT	986 - 1002<br>1079 - 1099	114	NM_214157.1	[65]
HADH	**F:** GCCATCGTGGAGAACCTGAA<br>**R:** GAAATGGAGCCCGGCAAATC	461 - 480<br>600 - 619	159	NM_214331.1	[64]
PGC1α	**F:** GATGTGTCGCCTTCTTGTTC<br>**R:** CATCCTTTGGGGTCTTTGAG	1629 - 1648<br>1702 - 1721	93	NM_213963.2	[66]
LPL	**F:** CCCTATACAAGAGGGAACCGGAT<br>**R:** CCGCCATCCAGTCGATAAACGT	448 - 470<br>564 - 580	138	NM_214286.1	[61]
CD36	**F:** CTGGTGCTGTCATTGGAGCAGT<br>**R:** CTGTCTGTAAACTTCCGTGCCTGTT	443 - 464<br>579 - 603	161	NM_001044622.1	[61]
CPT1α	**F:** CAAGATGGGCATGAACGCTG<br>**R:** TGGAATGTTGGGGTTGGTGT	1406 – 1425<br>1531 - 1550	145	NM_001129805.1	[60]
β-Actin	**F:** CTGCGGCATCCACGAAACT<br>**R:** AGGGCCGTGATCTCCTTCTG	944 - 962<br>1071 - 1090	147	XM_003124280.5	[63]

[1]*FAS = fatty acid synthase*; *ACC = acetyl-CoA carboxylase*; *HSL = Hormone-sensitive lipase*; *PPARα = Peroxisome proliferator activated receptor alpha*; *SREBP-1 = sterol regulatory element-binding protein 1*; *HADH = Hydroxyacyl-CoA dehydrogenase*; *PGC1α = PPARγ coactivator 1 alpha*; *LPL = lipoprotein lipase*; *CD36 = cluster of differentiation 36 molecule*, *CPT1α = carnitine palmitoyltransferase 1 α.*

Table S2. The host, dilution, and supplier of primary and secondary antibodies for immunoblotting.

**Antibodies**	**Host**	**Dilution**	**Vendor**
Anti *carbamoyl phosphate synthetase 1 (CPS1)*	Rabbit	1:1000	Abcam, Cambridge, MA, # ab45956
Anti *ornithine carbamoyl transferase (OTC)*	Rabbit	1:1000	Millipore Sigma, St. Louis, MO, # AV41766
Anti *argininosuccinate synthase 1 (ASS1)*	Mouse	1:1000	Abcam, Cambridge, MA, # ab124465
Anti *arginase 1 (ARG 1)*	Rabbit	1:1000	Proteintech, Rosemont, IL, # 16001-1-AP
Anti *arginosuccinate lyase (ASL)*	Rabbit	1:1000	Abcam, Cambridge, MA, # ab97370
Anti *β-actin* (C4) (HRP)	Mouse	1:1000	Santa Cruz Biotechnology, Dallas, TX, # sc-47778
Anti *GAPDH* (GT239) (HRP)	Mouse	1:5000	Thermo Scientific, Rockford, IL, # MA5-31457
Anti rabbit *IgG* H&L (HRP)	Goat	1:2000	Abcam, Cambridge, MA, # ab205718
Anti mouse *IgG* H&L (HRP)	Goat	1:5000	Abcam, Cambridge, MA, # ab205719

Table S3. Weekly growth performance of nursery pigs fed with very low-protein diets containing isoleucine, valine or mix of both above NRC levels

Measurements[2]	Diets[1] PC	NC	HV	HI	HVI	SEM[3]	*p*-value
**BWG, kg**							
Week 1	2.49^{a}	1.35^{b}	2.07^{ac}	1.28^{b}	2.04$^{c\gamma}$	0.10	< 0.01
Week 2	3.50^{a}	1.52^{b}	1.64^{b}	1.62^{b}	2.90$^{c\gamma}$	0.16	< 0.01
Week 3	4.29^{a}	1.92^{bc}	1.77^{b}	2.40^{bc}	2.82$^{c\varepsilon}$	0.19	< 0.01
Week 4	5.06^{a}	2.72^{bc}	2.47^{b}	2.59^{b}	4.04$^{ac\varepsilon\tau}$	0.24	< 0.01
Week 5	5.15^{a}	2.67^{b}	3.16^{b}	2.87^{b}	3.44^{b}	0.22	< 0.01
**MFI, kg**							
Week 1	0.39	0.28	0.38	0.30$^{\phi}$	0.38	0.01	0.02
Week 2	0.65^{a}	0.39^{b}	0.49^{bc}	0.46^{b}	0.58^{ac}	0.02	< 0.01
Week 3	0.89^{a}	0.52^{b}	0.61^{bc}	0.61^{bc}	0.78^{ac}	0.03	< 0.01
Week 4	0.97^{a}	0.68^{b}	0.80^{ab}	0.69^{b}	0.90^{ab}	0.03	0.01
Week 5	1.16^{a}	0.71^{b}	0.84^{b}	0.67^{b}	0.93$^{\gamma\tau}$	0.04	< 0.01
**CFI, kg**							
Week1	2.74	2.18	2.67	2.09$^{\phi}$	2.66	0.08	0.02
Week 2	4.54^{a}	2.73^{b}	3.43^{bc}	3.24^{bc}	4.04^{ac}	0.15	< 0.01
Week 3	6.24^{a}	3.63^{b}	4.28^{bc}	4.27^{bc}	5.49^{ac}	0.23	< 0.01
Week 4	6.81^{a}	4.76^{b}	5.58^{ab}	4.86^{b}	6.29^{ab}	0.23	0.01
Week 5	8.11^{a}	4.96^{b}	5.88^{b}	4.72^{b}	6.52^{ab}	0.28	< 0.01
**CPI, kg**							
Week 1	0.53^{a}	0.29^{b}	0.36^{b}	0.29^{b}	0.36^{b}	0.02	< 0.01
Week 2	0.89^{a}	0.36^{b}	0.46^{bc}	0.44^{bc}	0.54^{c}	0.03	< 0.01
Week 3	1.20^{a}	0.45^{b}	0.54^{bc}	0.59^{bc}	0.69^{c}	0.05	< 0.01
Week 4	1.37^{a}	0.50^{b}	0.71$^{c\vartheta}$	0.67^{bc}	0.79^{c}	0.05	< 0.01
Week 5	1.56^{a}	0.56^{b}	0.74^{bc}	0.65^{bc}	0.80$^{c\theta}$	0.07	< 0.01
**G:F, kg/kg**							
Week 1	0.89	0.62	0.78	0.67	0.80	0.03	0.09
Week 2	0.77^{a}	0.53^{bc}	0.49^{c}	0.50^{c}	0.68$^{a\theta}$	0.03	< 0.01
Week 3	0.69^{a}	0.54^{ab}	0.45^{b}	0.56^{ab}	0.49^{b}	0.02	< 0.01
Week 4	0.75	0.58	0.54	0.54	0.61	0.03	0.15
Week 5	0.64	0.53	0.53	0.64	0.53	0.03	0.49
**G:P, kg/kg**							
Week 1	4.57	4.54	5.76	4.79	5.88	0.24	0.22
Week 2	3.96^{ab}	3.89^{a}	3.64^{a}	3.72^{a}	5.01$^{b\theta}$	0.14	< 0.01
Week 3	3.57	4.42	3.45	4.09	3.90	0.14	0.17
Week 4	3.70	5.29	4.29	3.91	4.86	0.24	0.19
Week 5	3.33	4.46	4.30	4.61	4.28	0.22	0.32

[1]PC: positive control, standard protein diet; NC: negative control, very low protein diet containing first four limiting amino acids (*i.e.*, lysine, methionine, threonine, and tryptophan) at NRC levels; HV: NC containing valine (Val) above NRC level; HI: NC containing isoleucine (Ile) above NRC level; HVI: NC containing both Val and Ile above NRC level. The values are means, n=8. The *p*-values for the overall model effect for diet, week and diet × week for BWG were < 0.01, < 0.01 and < 0.01, for MFI were < 0.01, < 0.01 and < 0.01, for

CFI were < 0.01, < 0.01 and < 0.01, for CPI were < 0.01, < 0.01 and < 0.01, for G:F were < 0.01, < 0.01 and 0.22, and for G:P were < 0.01, < 0.01 and 0.15 respectively. [2]BWG: body weight gain; MFI: mean feed intake; CFI: cumulative feed intake; CPI: cumulative protein intake; G:F: gain:feed ratio; G:P: gain:protein ratio. [3]SEM: standard error of the mean. [a,b,c] Within each row, the values with different superscript letter(s) are different ($p \leq 0.05$). $^{\gamma}$ $p \leq 0.1$ HVI vs. PC, $^{\theta}$ $p \leq 0.1$ HVI vs. NC, $^{\varepsilon}$ $p \leq 0.1$ HVI vs. HV, $^{\tau}$ $p \leq 0.1$ HVI vs. HI, $^{\phi}$ $p \leq 0.1$ HI vs. PC, $^{\vartheta}$ $p \leq 0.1$ HV vs. NC.

Supplementary Figures

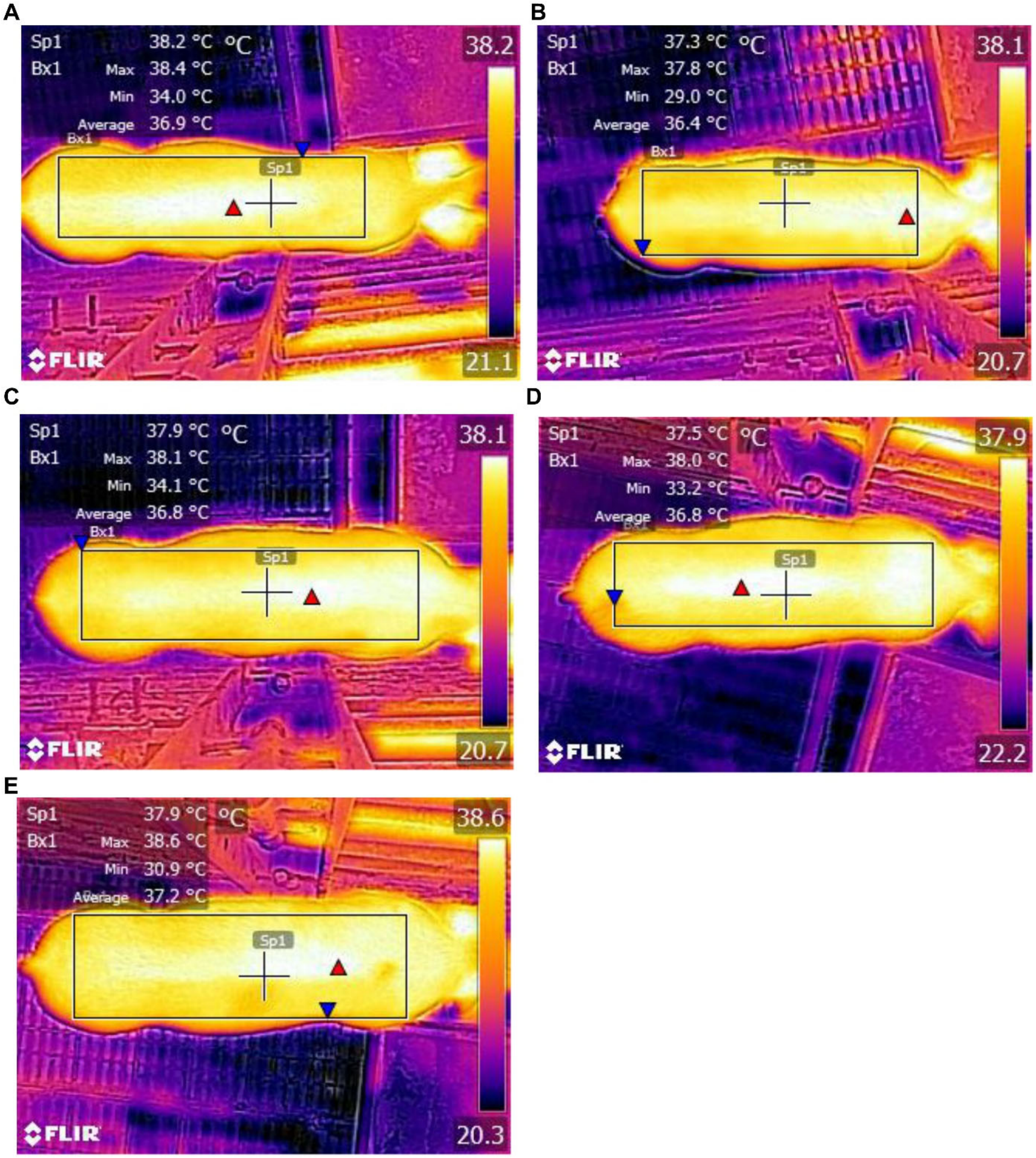

Figure S1. Representative thermal images of dietary group. (**A**) PC: positive control, standard protein diet, (**B**) NC: negative control, very low protein diet containing first four limiting amino acids (*i.e.*, lysine, methionine, threonine, and tryptophan) at NRC levels; (**C**) HV: NC containing Val above NRC level; (**D**) HI: NC containing Ile above NRC level; (**E**) HVI: NC containing both Val and Ile above NRC level. The minimum and maximum temperature spots in the rectangular box are shown by the blue and red triangles, respectively

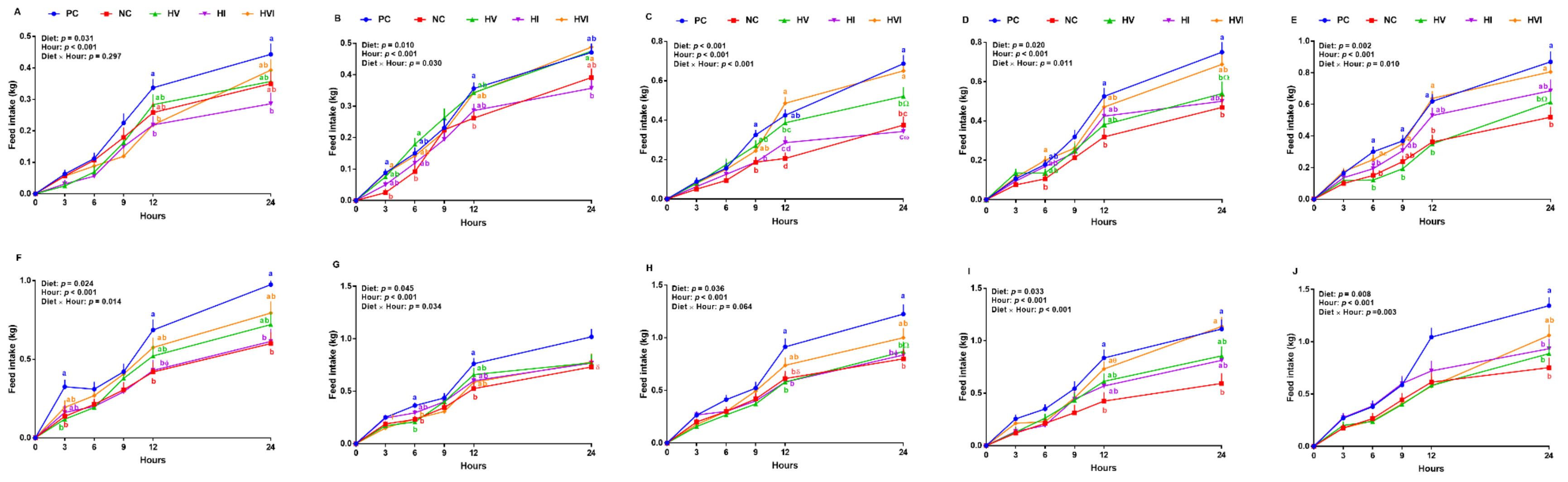

Figure S2. Feed intake of nursery pigs fed with very low protein diets containing isoleucine (Ile), valine (Val) or mix of both above NRC levels at (**A**) day 4, (**B**) day 7, (**C**) day 11 (**D**) day 14, (**E**) day 18, (**F**) day 21, (**G**) day 25, (**H**) day 28, (**I**) day 32, (**J**) day 35. PC: positive control, standard protein diet; NC: negative control, very low protein diet containing first four limiting amino acids (*i.e.*, lysine, methionine, threonine, and tryptophan) at NRC levels; HV: NC containing Val above NRC level; HI: NC containing Ile above NRC level; HVI: NC containing both Val and Ile above NRC level. The values are means ± standard error of the mean. n=8. ^{a,b,c,d,ab,bc,cd} the means with different superscript letter(s) at each time point are different ($p \leq 0.05$). $^{\delta}$ $p \leq 0.1$ PC vs. NC, $^{\Omega}$ $p \leq 0.1$ HV vs. PC, $^{\gamma}$ $p \leq 0.1$ HVI vs. PC, $^{\phi}$ $p \leq 0.1$ HI vs. PC, $^{\varepsilon}$ $p \leq 0.1$ HVI vs. HV, $^{\omega}$ $p \leq 0.1$ HV vs. HI

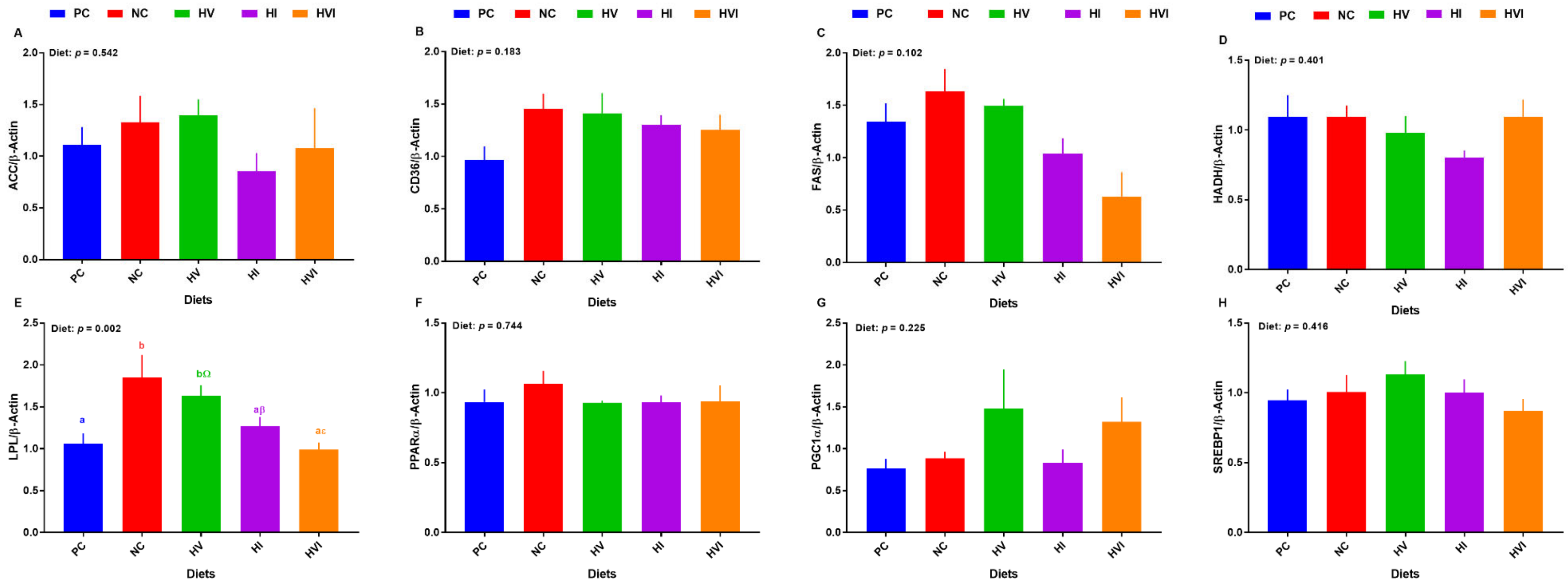

Figure S3. mRNA abundance of lipid metabolism markers in subcutaneous adipose tissue of nursery pigs fed with very low protein diets containing isoleucine (Ile), valine (Val) or mix of both above NRC levels. (**A**) *acetyl-CoA carboxylase alpha (ACC)*, (**B**) *cluster of differentiation 36 molecule (CD36)*, (**C**) *fatty acid synthase (FAS)*, (**D**) *hydroxyacyl-CoA dehydrogenase (HADH)*, (**E**) *lipoprotein lipase (LPL)*, (**F**) *peroxisome proliferator activated receptor alpha (PPARα)*, (**G**) *PPARγ coactivator 1 alpha (PGC1α)*, (**H**) *sterol regulatory element-binding protein 1 (SREBP-1)*. PC: positive control, standard protein diet; NC: negative control, very low protein diet containing first four limiting amino acids (i.e., lysine, methionine, threonine, and tryptophan) at NRC levels; HV: NC containing Val above NRC level; HI: NC containing Ile above NRC level; HVI: NC containing both Val and Ile above NRC level. The values are means ± standard error of the mean. n= 8. [a,b,ab] Among groups, the means with different superscript letter(s) are different ($p \leq 0.05$). $^{\Omega}\, p \leq 0.1$ HV vs. PC, $^{\beta}\, p \leq 0.1$ HI vs. NC, $^{\varepsilon}\, p \leq 0.1$ HVI vs. HV.
